# Behavioral and neuronal extracellular vesicle biomarkers associated with nicotine’s enhancement of the reinforcing strength of cocaine in female and male monkeys

**DOI:** 10.1016/j.addicn.2024.100151

**Published:** 2024-02-15

**Authors:** Mia I. Allen, Bernard N. Johnson, Ashish Kumar, Yixin Su, Sangeeta Singh, Gagan Deep, Michael A. Nader

**Affiliations:** a Department of Translational Neuroscience, Wake Forest University School of Medicine, Winston-Salem, NC, United States; b Center for Addiction Research, Wake Forest University School of Medicine, Winston-Salem, NC, United States; c Department of Cancer Biology, Wake Forest University School of Medicine, Winston-Salem, NC, United States; d J Paul Sticht Center for Healthy Aging and Alzheimer’s Prevention, School of Medicine, Wake Forest University, Winston-Salem, NC, United States; e Department of Cancer Biology, School of Medicine, Wake Forest University, Winston-Salem, NC, United States; f Atrium Health Wake Forest Baptist Comprehensive Cancer Center, Winston-Salem, NC, United States; g Department of Radiology, Wake Forest University School of Medicine, Winston-Salem, NC, United States

**Keywords:** Cocaine use disorder, Nicotine, Self-administration, Reinforcing strength, Drug choice, Impulsive Choice, Nonhuman Primates, Extracellular vesicles

## Abstract

While the majority of people with cocaine use disorders (CUD) also co-use tobacco/nicotine, most preclinical cocaine research does not include nicotine. The present study examined nicotine and cocaine co-use under several conditions of intravenous drug self-administration in monkeys, as well as potential peripheral biomarkers associated with co-use. In Experiment 1, male rhesus monkeys (*N* = 3) self-administered cocaine (0.001–0.1 mg/kg/injection) alone and with nicotine (0.01–0.03 mg/kg/injection) under a progressive-ratio schedule of reinforcement. When nicotine was added to cocaine, there was a significant leftward/upward shift in the number of injections received. In Experiment 2, socially housed female and male cynomolgus monkeys (*N* = 14) self-administered cocaine under a concurrent drug-vs-food choice schedule of reinforcement. Adding nicotine to the cocaine solution shifted the cocaine dose-response curves to the left, with more robust shifts noted in the female animals. There was no evidence of social rank differences. To assess reinforcing strength, delays were added to the presentation of drug; the co-use of nicotine and cocaine required significantly longer delays to decrease drug choice, compared with cocaine alone. Blood samples obtained post-session were used to analyze concentrations of neuronally derived small extracellular vesicles (NDE); significant differences in NDE profile were observed for kappa-opioid receptors when nicotine and cocaine were co-used compared with each drug alone and controls. These results suggest that drug interactions involving the co-use of nicotine and cocaine are not simply changing potency, but rather resulting in changes in reinforcing strength that should be utilized to better understand the neuropharmacology of CUD and the evaluation of potential treatments.

## Introduction

In 2018 more than one in five fatal drug overdoses involved cocaine and approximately 18.1 million people worldwide used cocaine [1,2]. Recent estimates indicate that more than 2.0 million Americans reported current cocaine use and the cost of substance use disorders in the United States alone is estimated at >$800 billion [3,4]. Of clinical significance, cocaine use is increasing and is currently the second leading cause of overdose deaths associated with illicit drug use [5]. While there are clinically effective pharmacotherapies available for opioid, nicotine, and alcohol use disorders, there are no FDA-approved treatments for cocaine use disorders (CUD) despite decades of research [6–10]. Given this, there remains a vital need for further clinical and preclinical research aiming to identify novel pharmacotherapies for CUD [11].

In the clinical setting, individuals who use cocaine have high rates of co-morbid cigarette smoking and estimates suggest that up to 80 % of individuals who use cocaine also co-use nicotine [12]. Studies have also shown that in individuals with CUD, nicotine/tobacco use is associated with earlier initiation of cocaine use, increased legal problems, more severe cocaine dependence, and poorer treatment outcomes [13,14]. Furthermore, severity of nicotine dependence is associated with greater number of positive drug screens for cocaine and poorer outcomes during treatment for CUD [13,15–18]. Taken together, these studies suggest that there may be significant deleterious effects associated with the co-use of nicotine and cocaine across multiple clinically relevant measures of treatment outcomes. Although the majority of individuals with CUD are polysubstance users, the inclusion of other commonly co-used substances, such as nicotine, in preclinical models of CUD is infrequent. Thus, a gap in the literature and the primary focus of the present study, is how nicotine influences cocaine self-administration.

Despite the dearth of preclinical research examining cocaine and nicotine co-use, two previous studies have examined this topic. In one study [19], male rhesus monkeys self-administered cocaine under a second-order fixed-ratio (FR) 2, variable-ratio 16 schedule of reinforcement and the inclusion of nicotine resulted in significant increases in rates of responding and in the number of injections earned. Freeman and Woolverton [20] studied nicotine and cocaine co-use, using a progressive-ratio (PR) schedule of reinforcement, to measures of reinforcing strength. In that study, they found that in four out of five rhesus monkeys, adding nicotine to cocaine shifted the cocaine dose-response curve to the left, but peak break points, the primary measure of reinforcing strength, did not increase. These findings suggest co-use of nicotine increased the potency of cocaine, but not the reinforcing strength.

One goal of the present study was to replicate the Freeman and Woolverton [20] study in rhesus monkeys self-administering cocaine, with and without nicotine, under a PR schedule of reinforcement. This replication is important because the parameters used in the Freeman and Woolverton study varied from the parameters used in our laboratory. For example, Freeman and Woolverton [20] incorporated 20-hour sessions compared with 4-hour sessions in the present study, as well as different fixed-ratio values and limited-hold conditions. Given that it has been argued that these parametric differences can affect the outcomes of the study [21], Experiment 1 aimed to determine if under a PR schedule of reinforcement, our findings correspond to those of Freeman and Woolverton.

In addition to replicating the Freeman and Woolverton study [20], we aimed to extend this characterization of the reinforcing strength of cocaine and nicotine co-use to responding under a concurrent drug vs. food choice schedule of reinforcement. Using a concurrent choice paradigm, three relevant independent variables were studied: social rank, sex, and the effect of implementing delays on cocaine and cocaine + nicotine choice. As it relates to social rank, monkeys living in social groups form hierarchies that are linear and transitory and these social hierarchies influence behavior and brain function [22–24]. These social hierarchies can be conceptualized as a continuum between chronic social stress and environmental enrichment. In fact, subordinate males who experience chronic exposure to social stressors self-administer cocaine at higher rates and have greater intakes when compared to dominant monkeys. In females, social rank also influenced vulnerable to cocaine reinforcement [25–27]. Thus, variables related to the organism such as sex and social rank appear to be crucial factors in influencing drug reinforcement [28]. Despite this, no previous work to our knowledge has examined if sex and/or social rank influence cocaine and nicotine co-use.

A third goal was to identify peripheral biomarkers, neuronally derived small extracellular vesicles (NDEs) associated with cocaine and nicotine co-use [29]. In the past few years, these lipid membrane-bound small extracellular vesicles (sEVs) have been extensively studied as a less-invasive tool to assess specific molecular biomarkers associated with various neurological disorders, including substance use disorders [30–34]. These vesicles are present in all biofluids and are loaded with cargo that could relate to the cell of origin and their physiologic/metabolic state. For example, plasma sEV reflected a similar change in the expression of glutamate receptor with aging as in the brain tissue [35]. We also recently reported that neurodegeneration (e.g., NfL and α-synuclein) and neuroinflammation biomarkers in brain cells-derived sEV in plasma closely correlated with a decrease in the gray matter volume of specific lobes of the brain measured by magnetic resonance imaging (MRI) in cynomolgus monkeys self-administrating oxycodone [36]. In the present study, neurally derived exosomes (NDEs) were characterized for the surface expression of several key molecular players involved in cocaine and/or nicotine’s pharmacological actions. We examined kappa opioid receptors (KOR), D2 dopamine receptors (D2DR), alpha 6 (α6R) and 7 (α7R) nicotinic receptors. The D2DR was selected as a target due to the fact that both nicotine and cocaine increase dopaminergic activity in brain regions such as the ventral tegmental area and nucleus accumbens; brain regions implicated in the facilitation of reinforcement [37,38]. In addition, clinical data suggest that nicotine enhanced and mecamylamine, a nicotine acetylcholine receptor antagonist, decreased cue-induced cocaine craving [39, 40]. Moreover, chronic cocaine and nicotine have been shown to reduce D2 receptor densities in brain regions such as the ventral striatum, caudate nucleus, and putamen, and low D2DR availability has been associated with higher rates of cocaine self-administration [41–45]. Thus, it’s possible that the co-use of cocaine and nicotine may result in greater reductions in the D2DR when compared to either drug alone which may further perpetuate drug self-administration [46]. A better understanding of the effect of cocaine with and without co-use of nicotine, on the expression of these key players (KOR, D2DR, α6R and α7R) in NDEs, could lead to development of novel blood-based biomarkers.

Negative reinforcement, mediated in part by the KOR and its endogenous ligand dynorphin, is hypothesized to play a role in substance use disorders and these aspects of drug-use likely perpetuate the transition from social drug use to compulsive drug use [47,48]. Rodent work has shown that cocaine exposure upregulated the KOR system which, in turn, increased susceptibility to stress-induced reinstatement [47,49–52]. Moreover, PET studies conducted in human subjects have demonstrated that following a 3-day cocaine binge, there were significant reductions in KOR availability in the striatum, caudate nucleus, putamen, and several other brain regions in the cortex [53]. In addition, studies have demonstrated that systemic administration of the KOR agonist U50488 reinstated nicotine self-administration in a dose-dependent manner in rats [54]. Given that the KOR seems to play an important role in drug self-administration this receptor was also selected as a target for the third goal of this study. Finally, some other potential targets that may modulate nicotine’s effect on cocaine reinforcement are the α6R and α7R nicotinic receptors. Previous studies have shown that α6R and α7R mediate the effects of nicotine on the mesolimbic dopamine pathway and induce DA release in the nucleus accumbens [55,56]. Moreover, cocaine is a weak inhibitor of nicotinic acetylcholine receptors, specifically the α6R, and this antagonism is thought to modulate dopamine release in the striatum and influence the reinforcing effects of cocaine [57,58]. The mechanisms by which nicotine enhances cocaine reinforcement is not well understood and data from NDEs may shed light on this question. It is important to note that, to our knowledge, no previous work has directly investigated the involvement of KOR, α6R and α7R on cocaine and nicotine co-use. In addition, identifying peripheral biomarkers associated with cocaine and nicotine co-use may help inform treatment selection for individualized care in the clinical population [59].

## Methods

### Animals

For the PR self-administration study (Experiment 1), subjects included 3 adult male single-housed rhesus monkeys (*Macaca mulatta*). For Experiment 2, 14 socially housed cynomolgus monkeys (6 females and 8 males) were used in the drug-food choice procedure and *n* = 10 of them (3 females and 7 males) were also used to examine the effects of nicotine co-use in delay discounting studies. All animals had at least 2+ years of cocaine self-administration history under various schedules of reinforcement. In Experiment 3, monkeys from Exp. 2, plus 6 cynomolgus monkeys who only self-administered nicotine (3/sex) and 10 drug-naïve cynomolgus monkeys (5/sex) were used. Blood samples were acquired, 43 total (18 male and 25 female samples), immediately after experimental sessions. The ages of the male rhesus monkeys ranged from 15 to 18 years and they weighed between 9.1–12.4 mg/kg. The males cynomolgus monkeys were between 4 and 13 years old and weighed between 5.6–8.6 mg/kg while the female cynomolgus monkeys were between 10 and 16 years old and weighted between 2.8–4.2 mg/kg. Moreover, social rank was determined according to the outcomes of agonistic encounters as described previously [41]. Monkeys in all experiments were housed in a temperature- and humidity-controlled room maintained on a 14-hour light/10-hour dark cycle (lights on between 6:00 AM and 8:00 PM) in stainless-steel cages with *ad-libitum* access to water. They were fed sufficient standard laboratory chow (Purina Lab-Diet 5045, St Louis, Missouri, USA) to maintain healthy body weights slightly below free-feeding weights and enriched daily with fresh fruits or vegetables. The menstrual cycle in all females was monitored daily. Environmental enrichment was provided as outlined in the Animal Care and Use Committee of Wake Forest University Non-Human Primate Environmental Enrichment Plan, including chew toys, mirrors, music, and foraging feeders. Animal housing, handling, and experimental protocols were performed per the 2011 National Research Council *Guidelines for the Care and Use of Mammals in Neuroscience and Behavioral Research* and were approved by the Animal Care and Use Committee of Wake Forest University.

### Catheter implantation

For the self-administration studies, monkeys were surgically implanted with a chronic indwelling intravenous catheter and subcutaneous vascular access port (VAP) under sterile conditions. Details of this surgery can be found in previously published work [60]. All animals were given at least one week to recover from the surgery before experimental sessions began.

### Apparatus

All monkeys, fitted with aluminum collars, were trained to sit in a primate restraint chair (Primate Products, Redwood City, CA). Behavioral experiments were conducted in ventilated, sound-attenuating primate chambers (1.5 × 0.76 × 0.76 m; Med Associates, St. Albans, VT). During each session, white noise was also played to mask any potentially obstructing sounds from outside the experimental room. Inside each chamber was an operant panel with horizontally aligned photo-optic switches (Model 117–1007; Stewart Ergonomics, Inc., Furlong, PA) that were positioned within reach of the monkey seated in a primate chair, and above each switch were stimulus lights. A food receptacle, located in the middle of the operant panel below the middle switch was connected to a pellet dispenser (Med Associates) on the top of the chamber with a Tygon tube for delivery of 1.0-g banana-flavored food pellets (Bio-Serv, Frenchtown, NJ). A peristaltic infusion pump (Cole-Parmer Instrument Co., Niles, IL) was also located on top of the chamber for intravenous drug delivery at a rate of approximately 1.5 mL per 10-sec. Illumination of a red light inside the chamber accompanied food presentations and drug injections and flashed during delays (~1-sec intervals).

Before each session, the area on the monkey’s back containing the VAP was prepared with an antiseptic povidone-iodine (PVP) scrub (Medline Industries, Mundelein, IL) followed by isopropyl alcohol (Fisher Scientific, Fair Lawn, NJ). The area was given a final preparation with PVP scrub before a 22-gauge Huber needle (Access Technologies) was inserted into the monkey’s port, connecting the catheter to the infusion pump on the chamber. Before the start of each session, the pump was operated for approximately 3-sec to fill each monkey’s port with saline or concentration of drug available for that session.

### Experiment 1

#### Effects nicotine on the reinforcing strength of cocaine under a progressive-ratio (PR) schedule of reinforcement.

Drug self-administration sessions began at approximately 8:00 AM each day and were conducted 5–7 days per week. Detailed methods have been described previously [61]. Briefly, daily behavioral sessions were comprised of a multiple schedule, in which the first component was a fixed-ratio (FR) 20 schedule of food availability, in which 20 responses on the right switch were required to deliver a food pellet. Each food reinforcer was followed by 5-sec time-out and the component ended after delivery of 10 banana-flavored food pellets or 5 min, whichever occurred first. Following a 5 min time-out (TO), the second component of the multiple schedule was a PR schedule of cocaine self-administration, available on the left switch. The first response requirement under the PR schedule was 20 and each available cocaine injection was determined by the formula of Richardson and Roberts [62]; there was a 10-sec TO after each injection. Sessions ended after a maximum of 30 injections, expiration of the 1 h limited hold (LH), or the 4-hr drug component elapsed (total session was 15,000 sec). The LH corresponds to the maximum time allotted for a monkey to receive a cocaine injection. For most sessions, the LH expired prior to the end of the 4-hr drug component and Table S1 depicts the average session time elapsed out of the total 15,000-sec session during each of the conditions tested. The number of injections earned in the session was the breakpoint (BP). After determining a cocaine dose-response curve (0.001–0.3 mg/kg/injection) in each monkey, 0.01–0.03 mg/kg/injection of nicotine was added to the cocaine solution, so that the BP for each cocaine dose was redetermined with each dose of nicotine. These doses of cocaine and nicotine correspond to the doses used in previous studies using rhesus macaques [19, 20]. Each drug dose was available for at least 3 sessions and until responding was deemed stable (mean percent drug choice ±20 % for 3 consecutive sessions, with no trends in choice behavior).

### Experiment 2

#### *Effects of nicotine on cocaine* vs *food choice*.

Monkeys were trained to respond under a concurrent FR food vs. cocaine choice schedule of reinforcement. All monkeys except F-8534 (FR 2) were trained to respond on the left or right response switches under an FR 30 schedule of reinforcement. Under these conditions, 30 consecutive responses on one switch always resulted in the delivery of a 1.0-g banana-flavored food pellet, while an FR 30 on the other switch resulted in activation of the infusion pump and an injection of cocaine (0.003–0.1 mg/kg/injection). Delivery of either reinforcer was accompanied by illumination of the red light above the corresponding switch for 10-sec and a 30-sec TO. Sessions began with two forced-choice trials or sampling trials of each contingency. During sampling trials, only one response switch was active signaled by the illumination of a white light above the switch to indicate availability. Completion of the FR resulted in the delivery of one stimulus (either a food pellet or an injection). Following the TO, the alternate response switch was illuminated, and completion of the FR resulted in the other stimulus. After the completion of both sampling trials, the schedule changed to a concurrent schedule, signaled by the illumination of the white light above each active response switch. To better assure contact with the contingencies and in order to limit the possibility of perseverative responding, a forced-choice was implemented during the session if a monkey selected the same response switch for 5 consecutive trials. Once the forced-choice was completed, the session returned to a concurrent schedule. Daily sessions, which typically occurred five days per week, ended after 30 free-choices or 60 min had elapsed, whichever occurred first. Most animals completed all available choices each session. Complete cocaine vs food choice dose-effect curves were determined for each monkey. Each cocaine (saline, 0.001–0.1 mg/kg/injection) dose was available for at least 3 sessions and until responding was deemed stable (mean percent drug choice did not vary ±20 % for 3 consecutive sessions, with no trends in choice behavior).

To examine the effects of nicotine on cocaine vs food choice, nicotine (0.01–0.056 mg/kg/injection) was added to the cocaine solution at two points based on the animal’s cocaine vs food choice dose-effect curves: the lowest cocaine dose at which a cocaine preference occurred (≥80 % cocaine choice) and the highest cocaine dose at which food was preferred (≤20 % cocaine choice). Each cocaine + nicotine solution was available for at least 3 sessions and until responding was deemed stable.

To further assess the effects of nicotine co-use on cocaine vs food choice, a delay discounting procedure was used. Under this procedure, a delay was added to the presentation of the preferred drug reinforcer, cocaine alone and cocaine + nicotine. This meant that following completion of the response requirement there was a delay before the reinforcer was presented to the animal. This delay was added to the lowest dose that resulted in >80 % drug choice. Initially, the delay value associated with both food and drug was 0-sec as described above. The delay value associated with 1.0-g banana-flavored food pellet remained at 0-sec throughout the experiment, while the delay associated with the drug reinforcer varied from 10- to 240-sec. During the delay, a red light above the response switch flashed on and off each second for the duration of the delay. Immediately following the delay, the drug dose was delivered followed by a 30-sec TO. As described above, sessions began with two forced-choice trials and sessions ended after 30 free-choices or 60 min elapsed, whichever occurred first. Delay values were kept constant for at least 3 consecutive sessions and until responding was deemed stable. Delay values were presented in ascending order to complete delay discounting curves for each monkey. In general, these delays were presented in the following order; 0, 10, 30, 60, 90, 120, 150, 180, 210, 240. However, in some animals, larger delay increments were used until percent drug choice was below 50 % (Fig. 4). Based on each monkey’s individual curve, an indifference point (IP) was interpolated as the delay value associated with the drug reinforcer that resulted in 50 % choice for food and drug. The dependent measure for this experiment was the IP value for cocaine and cocaine + nicotine, which can be used as a behavioral index of reinforcing strength.

### Experiment 3

#### Peripheral measures of KOR, dopamine receptor (D2DR), and nicotinic receptors (α6R and α7R) using NDE from plasma.

Blood samples were obtained from the femoral veins of awake monkeys who were seated in a primate restraint chair. These blood draws occurred immediately following self-administration sessions. All samples taken after cocaine self-administration (*n* = 14, 4 male and 10 female samples) and cocaine + nicotine self-administration (*n* = 18, 8 male and 10 female samples) occurred with animals responding under the drug-food choice procedure. Some of the animals used in the cocaine only group were used in the cocaine+nicotine group. However, the animals in the nicotine only group (*n* = 6, 3 female and 3 male samples) self-administered nicotine under a simple FR schedule of reinforcement (FR15 or 30). This was because nicotine did not function as a reinforcer in the drug vs. food choice procedure (Figs 2 and 3, points above 0 mg/kg/injection cocaine). The drug-naïve monkeys (*n* = 11, 4 male and 7 female samples) responded under a concurrent schedule of 1- vs. 3-food reinforcers; these monkeys had no previous exposure to cocaine or nicotine.

For all monkeys, total sEV (TE) were isolated from the plasma using a modified precipitation method described by us previously [30–32]. Briefly, plasma samples were centrifuged sequentially at 500 g for 5 min, 2000 g for 10 min, and 10,000 g for 30 min at 4 °C to remove any cell debris and large size vesicles. Finally, TE were isolated using ExoQuick (System Biosciences, Palo Alto, CA) and suspended in filtered Dulbecco’s Phosphate Buffered Saline (DPBS). NDE were isolated from TE using the surface marker synaptosomal-associated protein 25 (SNAP-25). For the isolation of SNAP-25 + NDE, TE were incubated with biotin-labeled SNAP-25 antibody (BioLegend, Cat. No. 836,308) overnight at 4 °C, with continuous mixing. Following antibody incubation, streptavidin-tagged agarose beads (ThermoFisher, Cat. No. 20,359) were added and incubated for 1 hour at room temperature while continuous mixing. Following incubation, samples were centrifuged at 2500x*g* for 3 min to remove the supernatant containing (SNAP-25) negative EVs. Beads were washed 3 times with washing buffer (PBS+0.1 % bovine serum albumin [BSA]) and SNAP-25+EVs (or NDE) were eluted in immunoglobulin G (IgG) elution buffer (ThermoFisher, Cat. No21004) for further characterization. The pH of the elution buffer was neutralized by adding 10 % v/v 1 M Tris (pH=9).

Size and concentration of all EV samples were analyzed with nano-particle tracking analysis (NTA) using Nanosight NS300 (Malvern Instruments, UK), as described previously [30–32]. A flow cytometry analysis was performed to analyze the expression of different markers on the surface of SNAP-25+EVs. All SNAP-25+EVs were incubated either alone or in specific combination with the following: Human KOR Alexa Fluor 647 Antibody (FAB3895R-100UG, R&D systems), Anti-D2DR/Dopamine D2 Receptor Antibody (B-10) PE (sc-5303 PE, Santa Cruz), Nicotinic Acetylcholine Receptor alpha 6/CHRNA6 Antibody (G-4) Alexa Fluor 647 (sc-376,966 AF647), and Nicotinic Acetylcholine Receptor alpha 7/CHRNA7 Antibody (319) PE (sc-58,607 PE, Santa Cruz) for 2 hour at room temperature and in the dark. Following antibody incubation, 60 μl of CellBrite steady 488 membrane labeling dye (CellBrite steady membrane staining Kit, Biotium, Fremont, CA), after 1:200 dilution in 0.1 μm filtered PBS, was added to the SNAP-25+EVs for 15 min incubation in the dark. Samples were diluted further to achieve an abort ratio of less than 10 %. EVs without membrane labeling dye were used to set the gate for dye positive EVs, and EVs with dye but without fluorescent antibodies were used to set the gate for PE/AF647 positive events. All samples were acquired on Cyto-Flex (Beckman Coulter Life Science, Indianapolis, IN) for 60-sec at a low flow rate. Filtered PBS was run between the samples.

### Drugs

(−)Cocaine HCl, supplied by the National Institute on Drug Abuse (Bethesda, MD), and nicotine bitartrate (Sigma-Aldrich, St. Louis, MO), were dissolved in sterile 0.9 % saline. Different doses were studied by changing the drug concentration. All drug doses are expressed as the salt form.

### Data analyses

For Experiment 1, the primary dependent variable was the number of drug injections as a function of dose, termed the break point. The effects of nicotine on cocaine breakpoints were analyzed at two doses: the cocaine dose that was at the peak of the dose-effect curve and a dose of cocaine that did not result in significantly more injections than saline. Note that doses were individually determined and not necessarily the same for each monkey in the analysis. One-way paired *t*-tests were used to determine which dose of cocaine did not differ from saline. Following this, additional one-way paired *t*-tests were performed to compare cocaine breakpoints when administered alone to cocaine breakpoints when administered in conjunction with nicotine. A one-tailed paired *t*- test was selected because we hypothesized that adding 0.01–0.03 mg/kg of nicotine to a cocaine solution would increase cocaine’s potency and reinforcing strength. Additionally, a one-way *t*-test was selected to maximize statistical power given the small sample size. A one-tailed paired *t*-test was also used to compare the number of injections received for saline to the number of injections received when nicotine alone (0.01–0.03 mg/kg/injection) was available. Finally, one-tailed paired *t*-tests were used to compare nicotine alone breakpoints to the breakpoints of the low cocaine dose + nicotine mixture and the peak cocaine dose+ nicotine mixture. Bonferroni’s correct was used for multiple comparisons. For each animal, the dose of nicotine (0.01–0.03 mg/kg/injection) that produced the greatest effect were used in the analysis.

For Experiment 2, the primary dependent variables were% drug choice and ED_50_ values on the ascending limb of the drug choice dose-response curves. ED_50_ values for cocaine and cocaine + nicotine were determined by calculating the linear values for dose of cocaine and cocaine + nicotine at which% drug choice was 50 %. These linear ED_50_ values where then analyzed using a mixed-effect analysis of variance (ANOVA) with ED_50_ values, sex, and social rank as the main factors. A significant ANOVA was followed by pairwise multiple comparisons (Holm-Sidak) post-hoc tests. Mauchly’s Test of Sphericity was used to indicate if the assumption of sphericity was violated and if it was, the Greenhouse-Geisser Correction was applied. Moreover, a mixed-effect ANOVA was run to compare percent choice for saline vs food to percent choice for nicotine alone vs food as a function of sex and rank (*n* = 6 females, *n* = 7 males). For this analysis, the dose of nicotine (0.01–0.056 mg/kg) that produced the greatest percent choice was used. The effect of delaying the preferred drug reinforcer was also analyzed and the primary dependent variable was percentage of total trials in which the drug reinforcer was chosen over food. When delays were implemented, an indifference point (IP) was calculated first for the lowest dose of cocaine that resulted in greater than 80 % choice and then for cocaine + nicotine at that same cocaine dose. The IP value was interpolated as the linear delay value that engendered 50 % choice of the delayed drug reinforcer. IP values for cocaine alone and cocaine + nicotine were compared using a two-way paired *t*-test across both rank and sex. A *t*-test was used because we were underpowered to evaluate sex or social rank differences in this analysis. In each monkey, the nicotine dose (0.01–0.03 mg/kg/injection) that resulted in the greatest effect, either an increase or decrease in percent choice, was used in the analysis. Significance was set at an alpha of 0.05 and all statistical tests were analyzed with SPSS.

For Experiment 3, the primary dependent variable was percentage positive NDEs for each marker of interest (KOR, D2DR, nicotinic α6R and α7R). The effect of condition (naïve, cocaine alone, nicotine alone, and cocaine + nicotine) and sex on each marker was analyzed using a two-way ANCOVA. The number of sessions an animal was on the dose of drug prior to the blood draw, as well as the total number of injections received on that dose of drug, were used as covariates in the analysis. Total cocaine intake and nicotine intake was also evaluated in reference to percentage positive NDEs, but no significant effects were found and thus, these variables were not included in the main analyses. If the two-way ANCOVA was not significant, the effect of condition on KOR, D2DR, nicotinic α6R and α7R NDE was analyzed across sex. A significant test was followed by pairwise multiple comparisons (Holm-Sidak) post-hoc tests. Significance was set at an alpha of 0.05 and all statistical tests were analyzed with SPSS.

## Results

### Experiment 1

#### Effects nicotine on the reinforcing strength of cocaine under a PR schedule of reinforcement.

**Fig. 1.**
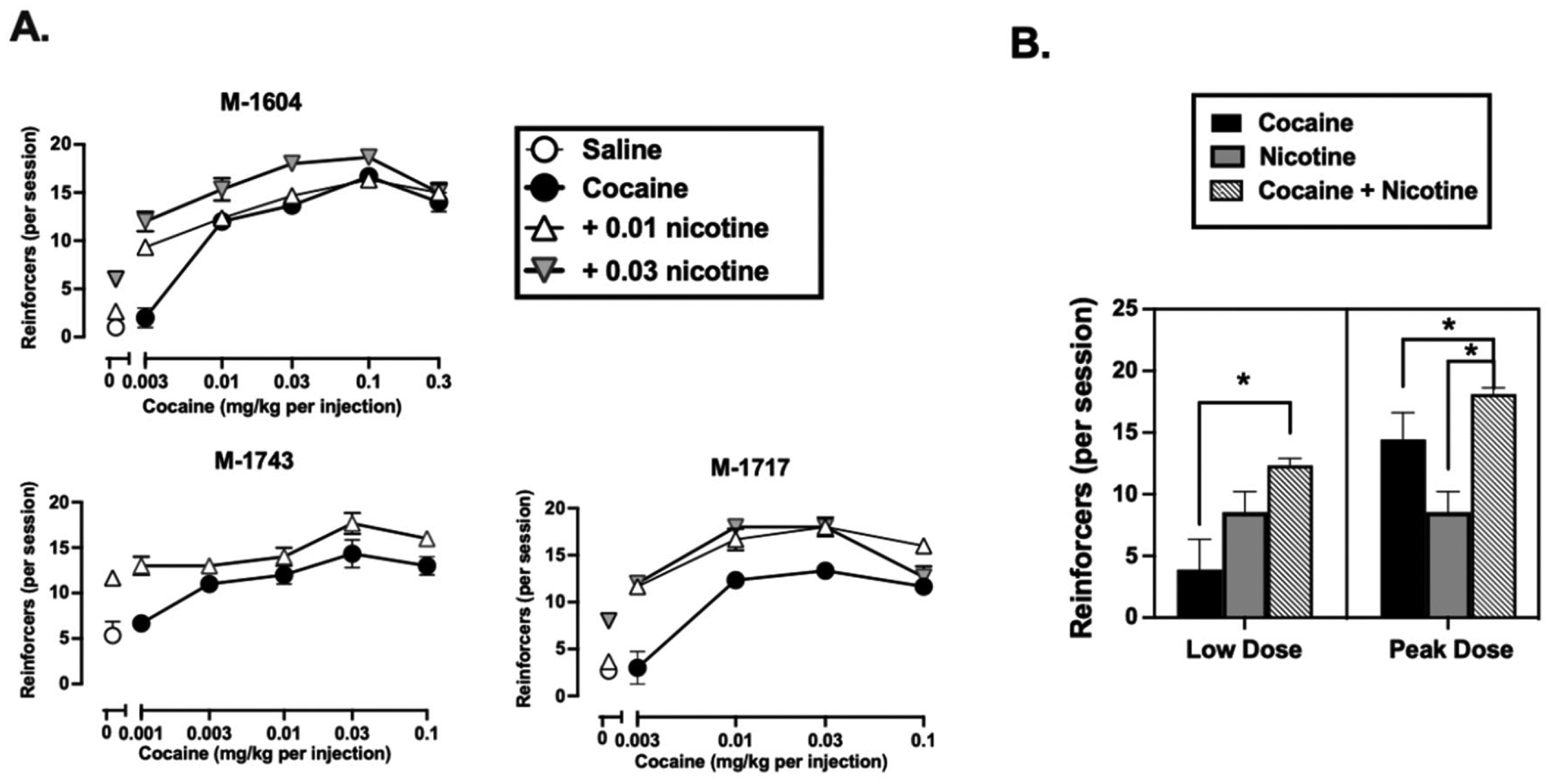
(A) Reinforcers earned (ordinate) as a function of cocaine dose (abscissa) in individual monkeys. Saline (open circles), cocaine (filled circles, 0.001–0.3 mg/kg/injection) and cocaine plus nicotine (open and shaded triangles, 0.01–0.03 mg/kg/injection) are shown as mean ± SD for the last three sessions. (B) Number of reinforcers earned when low-dose cocaine and peak cocaine (black bars) were studied and when nicotine (hatched bars) was added to the cocaine solution. Breakpoints for nicotine alone are also shown (gray bars). The doses of nicotine that resulted in the greatest effect are depicted in this bar graph. Each error bar represents the mean ± SEM. * *p* < 0.05.

Average session time (*sec*) for each condition where the total potential session time was 15,000 s can be seen in Table S1. The longest session times were associated with the peak break points for cocaine and cocaine + nicotine. Break point for cocaine increased in a dose-dependent manner in each monkey (Fig. 1A, filled circles). The doses of cocaine that did not differ significantly [*t*_(2)_ = −1.76, *p* = 0.110] from saline in terms of the number of injections received were 0.003 mg/kg/injection in M-1604 and M-1717 and 0.001 mg/kg/injection in M-1743 (Fig. 1A). When nicotine (0.01 mg/kg/injection in M-1743 and 0.03 mg/kg/injection in M-1604 and M-1717) was added to this low dose of cocaine there was a significant [*t*_(2)_ = −7.71, *p* = 0.008] increase in the number of reinforcers earned (Fig. 1A and 1B). When the peak BP for cocaine was studied (0.03 mg/kg/injection in M-1717, 0.03 mg/kg/injection in M-1743, and 0.1 mg/kg/injection in M-1604), the mean number of injections earned at these cocaine doses was 14.44 ± 2.17 (Fig. 1B). When nicotine (0.01–0.03 mg/kg/injection) was added to these cocaine doses there was a significant increase (18.11 ± 0.51) in the number of injections earned [*t*_(2)_ = 4.35, *p* = 0.038] (Fig. 1B). Nicotine alone (0.01 mg/kg/injection in M-1743 and 0.03 mg/kg/injection in M-1604 and M-1717) maintained a significantly greater number of drug injections when compared with saline [*t*_(2)_ = −10.61, *p* = 0.009]. Finally, nicotine alone resulted in significantly fewer injections when compared to the peak dose cocaine + nicotine combination (*p* = 0.019) but only marginally fewer injections when compared to the low dose cocaine + nicotine combination (*p* = 0.054) (Fig. 1B).

### Experiment 2

#### *Effects of nicotine on cocaine* vs *food choice*.

**Fig. 2.**
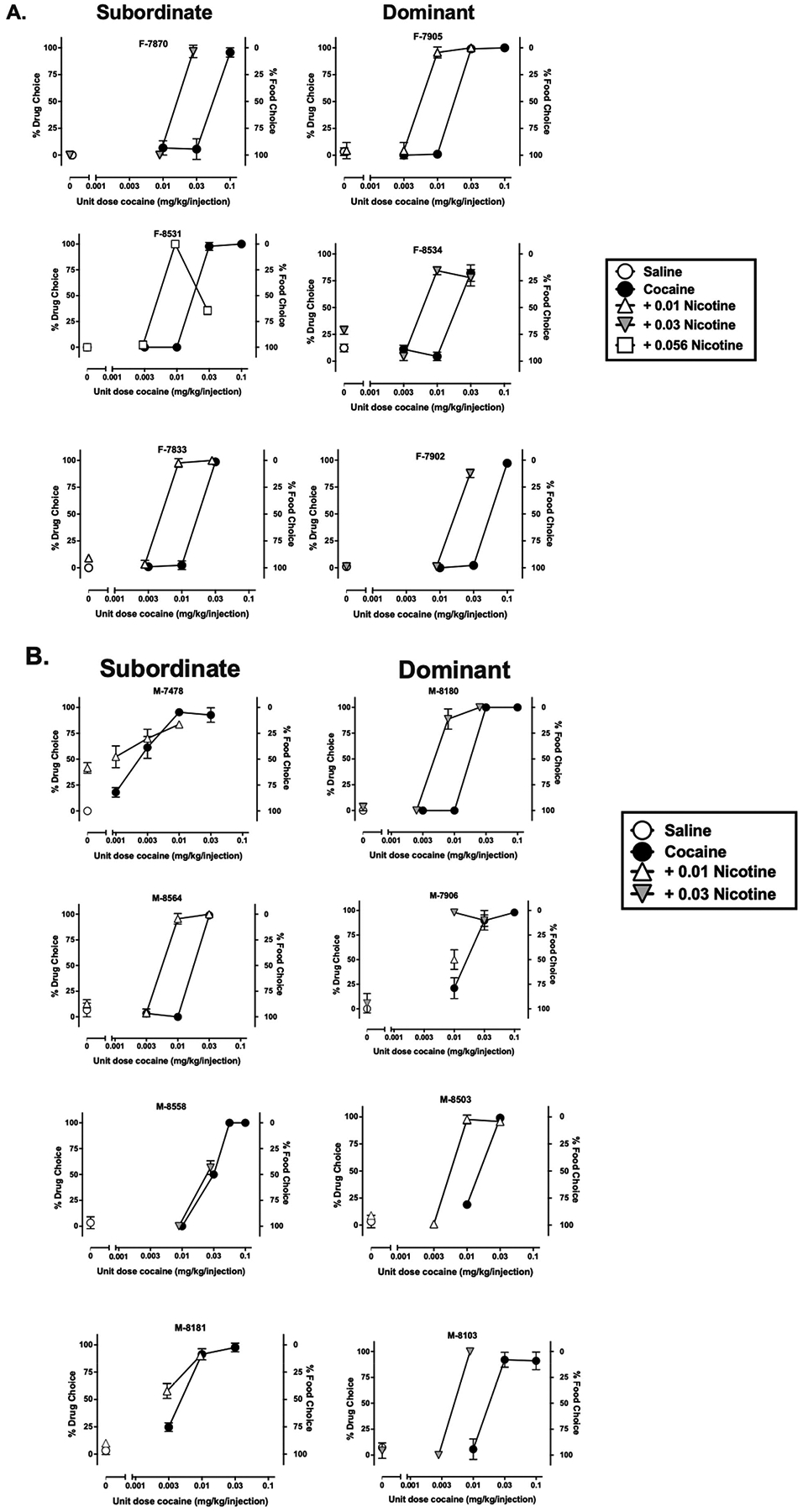
Percent drug choice (ordinate) as a function of cocaine dose (abscissa) in female (A) and male (B) monkeys. Subordinate animals are shown on the left and dominant animals are shown on the right. Data are mean ± SD of the last 3 sessions when choice was between food and cocaine (saline, 0.001–0.1 mg/kg, filled circles) or food and cocaine + nicotine (0.01–0.056 mg/kg, open and shaded triangles).

**Fig. 3.**
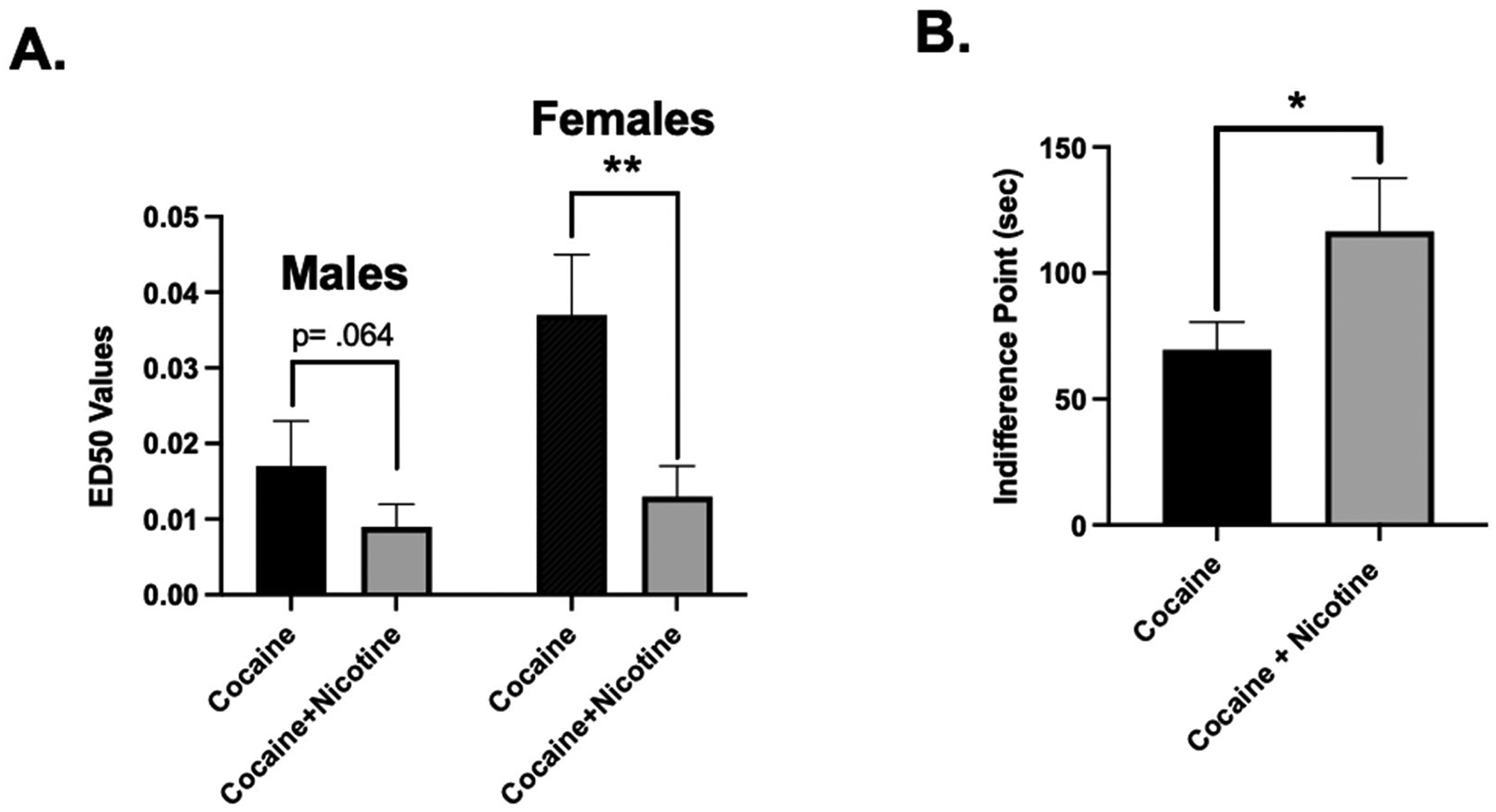
(A) ED50 values calculated from the ascending limbs of the dose-response curves in Fig. 2, for males and female monkeys. Bars represent ED_50_ values for cocaine alone (0.001–0.1 mg/kg, filled bars) and cocaine + nicotine (0.01–0.056 mg/kg, shaded bars). (B) Indifference points for cocaine alone (filled bar) and cocaine + nicotine (shaded bar) using delays as shown in Fig. 4. * *p* < 0.05; ** *p* < 0.01.

Cocaine choice increased relative to food reinforcement in a dose-dependent manner, such that low cocaine doses resulted in <20 % cocaine choice and higher doses >80 % cocaine choice in each female and male monkey (Fig. 2A and 2B, respectively; filled circles). Adding nicotine to the cocaine solution shifted the cocaine dose-response curve to the left in most monkeys (Fig. 2). Individual ED_50_ values can be found in the supplement for female and male monkeys (Tables S2 and S3 respectively). The mixed-effect ANOVA revealed a significant main effect of adding nicotine on cocaine ED_50_ values [*F*_(1,10)_ = 25.70, *p* < 0.001] and a significant interaction with sex [*F*_(1,10)_ = 5.24, *p* = 0.045]. *Post-hoc* analyses of the interaction found that females had significantly [*F*_(1,10)_ = 24.37, *p* < 0.001] lower ED_50_ values with the addition of nicotine (0.01–0.056 mg/kg/injection) when compared to ED_50_ values with cocaine alone (0.009 ± 0.003 vs. 0.034 ± 0.007 mg/kg/injection, respectively), (Figs. 2 and 3). Males, on the other hand, only experienced a marginal [*F*_(1,10)_ = 4.35, *p* = 0.064] reduction in ED_50_ values with the addition of nicotine (0.01–0.056 mg/kg/injection) when compared to ED_50_ values with cocaine alone (0.009 ± 0.003 vs. 0.017 ± 0.006 mg/kg/injection, respectively). There were no significant differences between male and female monkeys with cocaine alone ED_50_ values or cocaine + nicotine ED_50_ values. Also, there was no significant interaction with social rank [*F*_(1,10)_ = 0.15, *p* = 0.709] or three-way interaction between ED_50_ values, sex and rank [*F*_(1,10)_ = 0.01, *p* = 0.92]. When given the choice between nicotine and food presentation, a mixed-effect ANOVA demonstrated that percent choice for nicotine was not different than percent choice for saline when food was available as an alternative [*F*_(1,9)_ = 1.81, *p* = 0.21], (Fig. 2). Sex and social rank were not significant factors in this analysis (*p*>0.05).

**Fig. 4.**
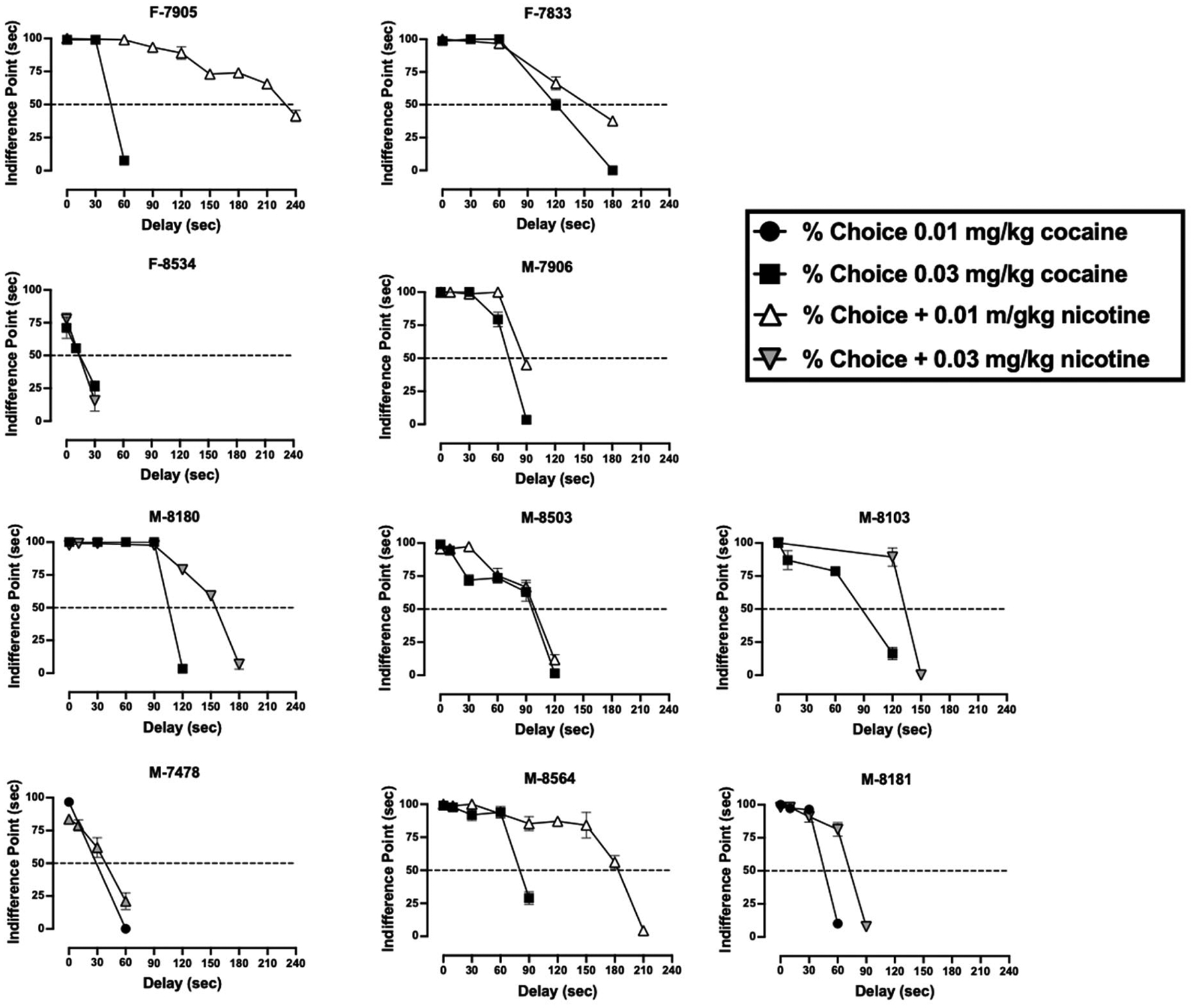
Indifference points (ordinate) as a function of delay (abscissa) in individual monkeys. Delays were studied on food vs. cocaine choice (filled symbols) and food vs. cocaine + nicotine choice (open and shaded triangles). Animal numbers starting with M are males and animal numbers starting with F are female animals. Each point represents the mean ± SD.

Delays were incorporated on the lowest drug doses that resulted in >80 % drug choice. For cocaine alone, the IP values ranged from 13.8 to 120.0 s and for cocaine and nicotine combinations, the IP values ranged from 13.3 to 229.3 (Fig. 4). Individual IP values can be found in the supplement (Table S3). When nicotine was added to cocaine, analyses revealed that the IP scores of cocaine + nicotine combinations (116.60 ± 66.77) were significantly [*t*_(9)_ = −2.63, *p* = 0.028] larger than the IP scores of cocaine alone (69.57 ± 34.66), (Figs. 3 and 4).

### Experiment 3

#### Peripheral measures of KOR, dopamine receptor (D2DR), and nicotinic receptors (α6R and α7R) using NDE from plasma.

**Fig. 5.**
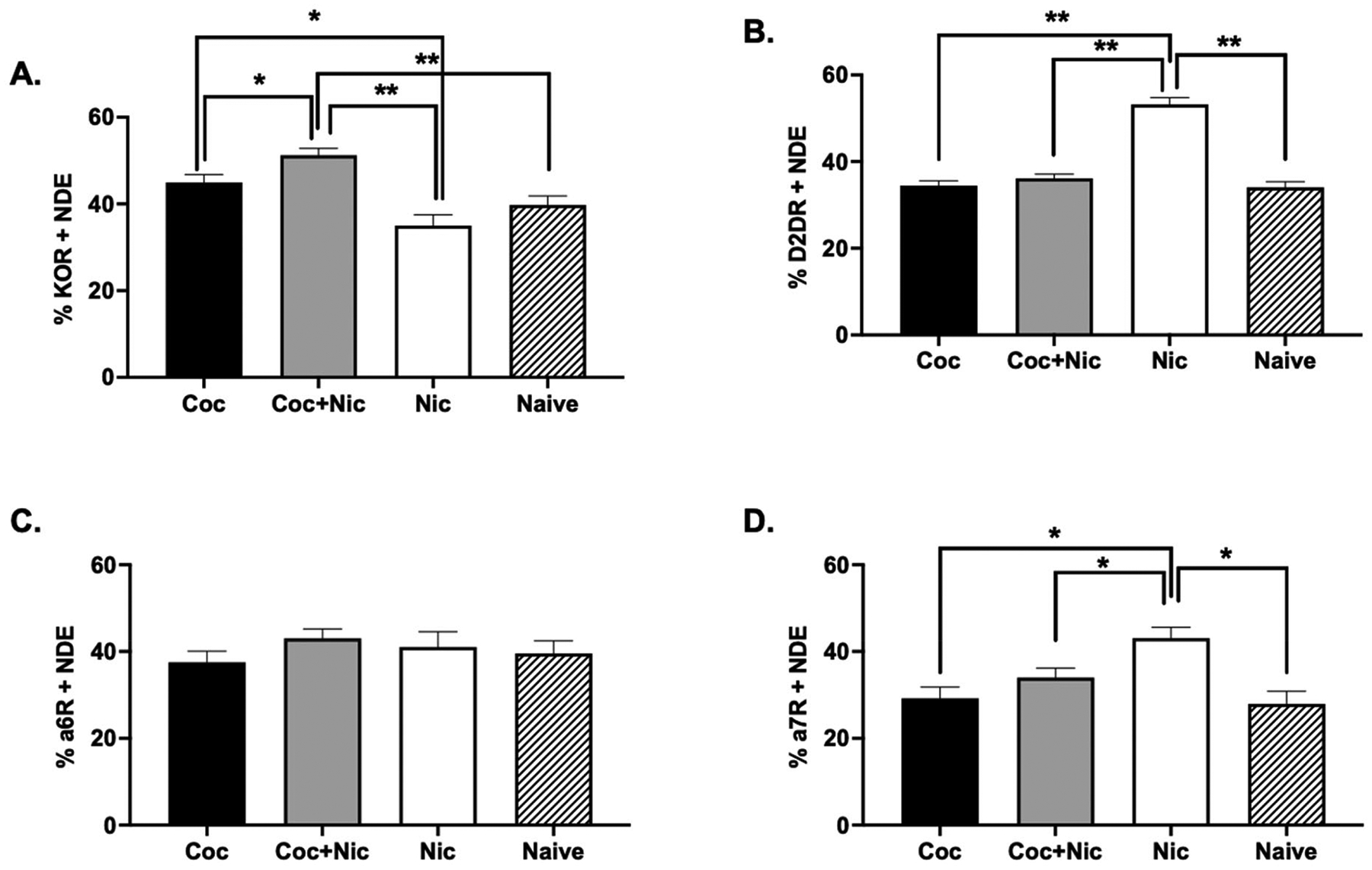
Percentage of NDEs (ordinates) for (**A**) KOR, (**B**) D2DR, (**C**) α6R (**C**) and (**D**) α7R obtained from blood samples taken after cocaine (filled bars), cocaine + nicotine (gray bars), nicotine (open bars), or food (hatched bars) self-administration. Each bar represents the mean ± SEM. * *p* < 0.05; ** *p* < 0.01.

In the cocaine-only group, each blood sample was taken after 11.64 (SD=14.90) days and after 123.57 (SD=160.73) injections of cocaine. In the cocaine+nictoine group each blood sample was taken after 11.06 (SD=7.99) days and after 160.88 (SD=137.95) injections of the drug combination. In the nicotine-only group, each blood sample was taken after 5 (SD=0) days and after 77.83 (SD=50.94) injections. Length of time on a drug dose and the number of injections received at that dose were not significant in any of the following analyses. NTA data showed that for all TE and NDE samples, the average size was below 200 nm (data not shown), suggesting the enrichment for sEV through our isolation method. The flow cytometry analyses of KOR+ NDE as a function of condition and sex revealed a significant main effect of condition [*F*_(3,38)_ = 12.09, *p* < 0.001], but no effect of sex or interaction between sex and condition (*p* > 0.05). *Post-hoc* analyses revealed that the samples taken after cocaine + nicotine co-administration expressed significantly higher KOR+ NDE (*M* = 51.25, SE = 1.55) when compared to the naïve samples (*M* = 39.79, SE = 2.07; *p* < 0.001), cocaine-alone samples (*M* = 44.96, SE = 1.83; *p* = 0.013), and nicotine-only samples (*M* = 35.01, SE = 2.49; *p* < 0.001) (Fig. 5A). There were no differences between the naïve samples and nicotine-only samples (*p* = 0.14) or naïve samples and cocaine-alone samples (*p* = 0.73). Cocaine alone also resulted in significantly higher KOR+ NDE when compared to nicotine alone (*p* = 0.003).

Statistical analyses of D2DR+ NDE found that there was a significant effect of condition [*F*_(3,38)_ = 38.95, *p* < 0.001] and sex [*F*_(1,38)_ = 5.34, *p* = 0.026], but no significant interaction (*p* = 0.052). Post-hoc analyses revealed that the nicotine-alone samples expressed significantly higher D2DR+ NDE (*M* = 53.17, SE = 1.58; *p* < 0.001) when compared to naïve (*M* = 34.07, SE = 1.31; *p* < 0.001), cocaine-alone (*M* = 34.42, SE = 1.16; *p* < 0.001), and cocaine + nicotine samples (*M* = 36.12, SE = 0.98; *p* < 0.001) (Fig. 5B). No other relationships were significant (*p* > 0.05). Moreover, female monkeys across condition expressed higher D2DR+ NDE (*M* = 40.90, SE = 0.79) when compared to males (*M* = 37.99, SE = 0.97; *p* = 0.026).

Analyses of α6R+ NDE across sex revealed no effect of condition [*F*_(3,42)_ = 1.01, *p* = 0.397] (Fig. 5C), while analyses of α7R+ NDE revealed a significant main effect of condition [*F*_(3,38)_ = 4.53, *p* = 0.008] and sex [*F*_(1,38)_ = 6.77, *p* = 0.013] but no interaction (*p* = 0.645). *Post-hoc* analyses revealed that the nicotine-alone samples had significantly higher α7R+ NDE (*M* = 43.04, SE = 2.54) when compared to the naïve samples (*M* = 27.91, SE = 2.94; *p* = 0.002), cocaine-alone samples (*M* = 29.22, SE = 2.60; *p* = 0.004), and cocaine + nicotine samples (*M* = 33.99, SE = 2.20; *p* = 0.037) (Fig. 5D). No other relationships were significant (*p* > 0.05). Furthermore, females (*M* = 37.21, SE = 1.78) had significantly higher α7R + NDE when compared to males (*M* = 29.86, SE = −2.17; *p* = 0.013).

## Discussion

The majority of people with CUD also co-use nicotine products [12]. Thus, the goal of the present set of studies was to examine, in nonhuman primate models, the effects of co-use of nicotine on the reinforcing strength of cocaine under several conditions in monkeys. In monkeys responding under a progressive-ratio schedule of reinforcement, the co-use of nicotine resulted in significant upward and leftward shifts in the dose-effect curves. In a second experiment using a concurrent drug vs. food choice paradigm, co-use of nicotine increased the potency of cocaine and shifted the cocaine dose-effect curve leftward in most animals. Although social rank did not influence the magnitude of these leftward shifts, the effect was statistically significant only in female animals. To better determine if the reinforcing strength of cocaine + nicotine was greater than cocaine alone, delays were implemented on drug choices. We found that the choice for the co-use of nicotine and cocaine self-administration required significantly longer delays to decrease drug choice compared with cocaine alone, confirming that the co-use of nicotine increased the reinforcing strength of cocaine. To examine potential mechanisms mediating this enhancement of cocaine reinforcement by nicotine, blood samples were taken post-session to identify potential peripheral biomarkers. We found significant differences in the KOR, D2DR, and α7R positive NDE in the plasma in samples taken following cocaine + nicotine self-administration compared with the other conditions. The fact that nicotine enhanced the reinforcing strength of cocaine and altered CNS markers related to reinforcement, these results highlight the need for preclinical models of CUD to include the co-use of nicotine when evaluating CNS mechanisms and potential interventions to decrease cocaine use.

In all the experiments described, models of reinforcing strength were used. As defined by Woolverton and Nader [29], these are models in which the reinforcing effects of a drug (or drugs) are measured in a quantitative (i.e., magnitude of reinforcing effects), rather than qualitative (i.e., response rates) manner. Since response rates are influenced by the direct effects of the drug on responding, schedules of reinforcement that can provide an uncontaminated index of response strength or resistance of behavior to change by various manipulations are considered models of reinforcing strength (or efficacy). Two of the more frequently used schedules of reinforcement assessing measures of reinforcing strength are progressive-ratio (PR) and concurrent choice schedules.

Although this study replicated the leftward shifts in the cocaine dose-effect curves with the addition of nicotine from the Freeman and Woolverton study [20], we also observed significant increases in break point for cocaine + nicotine compared with cocaine alone. In the clinical setting, this may suggest that cocaine has higher reinforcing strength in combination with nicotine when compared to cocaine alone. As mentioned previously, a potential reason for the discrepancy between this study and the Freeman and Woolverton [20] study may lie in methodological differences. One particularly important difference may be the timeout length between injections. For instance, in the Freeman and Woolverton study the inter-trial interval was 30-min while in this study it was 10-sec. It’s possible that the use of shorter timeouts between injections heightened nicotine’s ability to potentiate cocaine’s reinforcing strength, increased the reinforcing effects of nicotine alone, and/or influenced nicotine’s ability to attenuate the rate-decreasing effects of cocaine [63,64]. It’s also important to note that in the Freeman and Woolverton study, nicotine alone did not function as a reinforcer which may suggest that in this study the increase in cocaine’s reinforcing strength with the addition of nicotine may be, in part, due to nicotine having an additive effect of cocaine’s reinforcing strength [20].

In a second model of reinforcing strength, a concurrent choice procedure, we extended findings from the PR study and indicated an increase in the potency of cocaine to function as a reinforcer with the addition of nicotine. This effect was especially apparent in female monkeys and previous clinical and preclinical work suggests that this may be because of sex differences in sensitivity to the effects of nicotine. One study in humans demonstrated that women had a greater sensitivity to the subjective effects of intravenous nicotine when compared to men [65] and a preclinical study showed that female rats self-administered nicotine under a PR schedule to greater break points compared with males [66]. In general, these findings support the notion that women may be especially vulnerable to increases in cocaine reinforcement when also using nicotine-containing products. Given that, to our knowledge, no studies have investigated how sex influences the potentiation of cocaine choice with the addition of nicotine, future studies should elucidate the neural correlates that may make females more sensitive to nicotine’s effects on cocaine, as well as how sex may influence the ability of behavioral and pharmacological interventions to decrease the co-use of cocaine and nicotine. While previous work has demonstrated that cocaine choice varied as a function of social rank in monkeys, the animals used in this study did not show differences in cocaine or cocaine + nicotine choice as a function of rank [41]. One possible explanation for this is that rank-related differences in cocaine choice may dissipate following chronic cocaine self-administration. However, we think this hypothesis is unlikely because an earlier study showing social rank-related differences in male monkeys, involved monkeys with similar cocaine histories (2–5 years of cocaine self-administration prior to responding under the concurrent choice schedule of reinforcement) to the monkeys in the present study [67]. However, future work should still evaluate whether social rank influences nicotine-induced changes in cocaine choice in a population of animals with less extensive cocaine histories.

Although nicotine functioned as a reinforcer under a PR schedule of reinforcement, percent choice for nicotine under the concurrent drug vs. food choice procedure was not different from that of saline. This suggests that the presence of an alternative reinforcer may modulate the reinforcing effects of nicotine and reduce the probability that nicotine is self-administered intravenously. Unlike with the PR experiment, this finding supports the notion that nicotine’s ability to facilitate cocaine’s choice is not due to nicotine’s reinforcing effects. Woolverton and Nader (1990) noted that reinforcing efficacy (or strength) of a drug is not an immutable property of the drug but can be affected by environmental variables [68]. The present data suggest that in the context of an alternative non-drug reinforcer, the reinforcing strength of intravenous nicotine is not present. Importantly, though, these doses of nicotine appeared to enhance the reinforcing strength of cocaine.

Through examining the consequences of delaying drug choice on ongoing drug self-administration, we also demonstrated that adding nicotine to the cocaine solution significantly increased the delay needed to decrease drug choice compared with cocaine alone. Thus, as with data from the PR study, the co-use of nicotine enhanced the reinforcing strength of cocaine and required significantly larger interventions to decrease drug use. These results demonstrate that cocaine choice is less sensitive to behavioral interventions when nicotine is also self-administered. Clinically, this may suggest that behavioral interventions which are normally effective in a population that uses only cocaine may be less effective in a population that uses nicotine in conjunction with cocaine. Future studies need to extend these interventions to include pharmacological treatments. Furthermore, although we were underpowered to examine the effect of sex on delay discounting, future work needs to examine if there are sex differences in sensitivity to behavioral interventions when nicotine is used with cocaine.

To understand the effects of cocaine, nicotine, and cocaine + nicotine on key molecular biomarkers, we employed NDEs as surrogates to understand how drug condition affected KOR, D2DR, α6R and α7R expression in the CNS. In the past few years, numerous studies have shown the utility of NDE in plasma to assess specific biomarkers and molecular changes in the brain associated with various neurological disorders, as well as in assessing treatment responses [30–34,69–71]. In the present study, we observed significant changes in KOR-positive NDE in the cocaine + nicotine group compared to the other groups (i.e., cocaine alone, nicotine alone, drug-naïve controls). Moreover, there were differences in D2DR, and α7R-positive NDE in the nicotine only group when compared to all other groups.

Though it is tempting to suggest corresponding changes in the expression of these molecules in the CNS neurons, this conclusion will require further validation either through neuroimaging measures using specific PET tracers and/or direct confirmation of their expression in brain tissues. It is also important to note that the changes in NDEs in this study do not parallel published PET data on region-specific changes in these receptors. In particular, previous work has demonstrated that cocaine exposure reduced D2DR availability and KOR availability in brain regions like the striatum, caudate nucleus, and putamen [43,44, 53]. However, in the present study, we saw no differences in D2DR or KOR NDEs between cocaine-naïve monkeys and cocaine-experienced monkeys. These data suggests that, while the expression of various molecular biomarkers, basal as well as in response to cocaine ± nicotine, could be brain-region specific, our current methodology, based on a single brain specific surface marker (SNAP25), does not offer brain region specificity. Despite these limitations, identifying the changes in the surface expression of KOR-, D2DR-, and α7R-positive on NDEs in the blood is still important, as a less-invasive approaches to assessing the effects of cocaine+/−nicotine use on these biomarkers and would likely lead to implementation in clinical studies. The use of NDEs as biomarkers for substance use disorders is still in its infancy [72], so the significance of these data may become more apparent with additional data from other studies.

There are some additional limitations to these studies. With the PR schedule of reinforcement, it appears that a ceiling effect may have been evident in some monkeys, such that the break point only occurred because the 4 hour session timed out. However, based on recent findings in which PR parameters were manipulated using a within-subjects design [61], it is likely that a longer session length, without changing other parameters, would have increased the break point for both cocaine and cocaine + nicotine and not changed the overall conclusion for this experiment. As it relates to the cocaine vs. food choice studies, we could expand this paradigm to include studies involving reinstatement/relapse, as described by others [73]. More specifically, when saline is substituted for the drug of choice, responding is reallocated to food, so concurrent choice paradigms can be used to assess reinstatement, without extinguishing responding.

Another limitation was that in all studies, relatively short exposure to nicotine was examined. While all studies showed potentiation and enhancement of the reinforcing strength of cocaine, it is an empirical question as to whether tolerance would develop to the effects of nicotine and whether nicotine dependence would produce qualitatively and quantitively different outcomes. It is also possible that the psychostimulant effects of nicotine increased overall responding, rather than increased the reinforcing strength of cocaine. However, this seems unlikely since nicotine would also increase food choice, resulting in approximately 50 % choice, rather than shifts to the left. Future studies could examine this potential behavioral mechanism, by examining nicotine-induced changes in ongoing operant responding, using a multiple schedule of reinforcement with responding maintained by food in one component and nicotine self-administration in the other.

An additional limitation is that, in the clinical population, smokers tend to be habitual users and are often dependent on nicotine [74]. Thus, the findings of these studies should be interpreted with the caveat that chronic nicotine use with physical dependence could have differing effects on cocaine self-administration. Follow-up studies should be conducted to determine how nicotine dependent animals differ from non-dependent animals in terms of enhancing the reinforcing strength of cocaine. In addition, in the human condition, cocaine + nicotine are often used sequentially instead of concurrently and it is possible that the pattern of consumption influences the interaction between the two drugs. Also, in humans, nicotine is generally smoked and the pharmacokinetic and pharmacodynamic interactions between nicotine and cocaine may differ based on the route of administration [75]. Given this, future studies hope to extend these findings to a paradigm where the monkeys are given access to smokable nicotine and cocaine. Lastly, flow cytometry data are presented as percentage positive NDE for KOR, D2DR, α6R and α7R, but these results may not represent absolute quantification due to the nano size of EVs multiple factors, including EVs concentration, antibody type, free fluorochrome dye, type of fluorochrome, and smaller non EVs particles that can affect their precise quantification.

In conclusion, we found that the co-use of nicotine enhanced the potency and reinforcing strength of cocaine under several conditions in monkeys. We also noted that females showed greater effects of nicotine + cocaine compared with male monkeys. Finally, the ability to identify potential peripheral biomarkers associated with this enhancement in reinforcing strength may inform future research aiming to identify the neural correlates of cocaine + nicotine co-use. Overall, these data provide novel insights into the behavioral interactions between nicotine and cocaine and suggest that different treatment interventions for CUD may be required in a population that also uses nicotine.

## Supplementary Material

1

## Data Availability

Data will be made available on request.
