# Supplementary material for: Behavioral and neuronal extracellular vesicle biomarkers associated with nicotine’s enhancement of the reinforcing strength of cocaine in female and male monkeys
Source: Addict Neurosci. Author manuscript; Available in PMC 2024 Jun 21. (PMC11192513; doi:10.1016/j.addicn.2024.100151)
Supplement: 1 [file NIHMS2001150-supplement-1.docx]

**Supplementary Files:**

**Behavioral and Neuronal Extracellular Vesicle Biomarkers Associated with Nicotine Enhancement of the Reinforcing Strength of Cocaine in Female and Male Monkeys**

Mia I. Allen^1,2^, Bernard N. Johnson^1,2^, Ashish Kumar^3^, Yixin Su^3^, Sangeeta Singh^3^, Gagan Deep^2,3,4^, and Michael A. Nader^1,2,5^

^1^Department of Physiology and Pharmacology, Wake Forest University School of Medicine, Winston-Salem, North Carolina

^2^Center for Addiction Research, Wake Forest University School of Medicine, Winston-Salem, North Carolina

^3^Department of Internal Medicine, Wake Forest University School of Medicine, Winston-Salem, North Carolina

^4^J Paul Sticht Center for Healthy Aging and Alzheimer's Prevention, Wake Forest University, School of Medicine, Winston-Salem, North Carolina

^5^Department of Radiology, Wake Forest University School of Medicine, Winston-Salem, North Carolina

Address Correspondence to:

Michael A. Nader, Ph.D.

Department of Physiology and Pharmacology

Wake Forest University School of Medicine

NRC 546, Medical Center Blvd.

Winston-Salem, NC 27157-1083

PH: 336-713-7172

Fax: 336-713-7180

[mnader@wakehealth.edu](mailto:mnader@wakehealth.edu)

**Running Title:** Effects of nicotine on cocaine reinforcement in monkeys

**TABLE S1: Average Session Time (sec) for Each Condition.**

| **Condition** | **Average session time (sec) elapsed** |
| --- | --- |
| Saline | 5586.46 ± 1155.28 |
| Low dose cocaine alone | 5169.33 ± 233.04 |
| Low dose cocaine + nicotine | 6367.22 ± 1197.41 |
| Peak dose cocaine alone | 13446 ± 1449.29 |
| Peak dose cocaine + nicotine | 14551.45 ± 389.47 |
| Nicotine alone | 6000.11 ± 982.16 |

Maximum session length was 15000 sec. Each value is the mean ± SEM of the last 3 sessions in each condition (*n*=3).

**TABLE S2: ED50 Values for Individual Female Monkeys Responding Under Concurrent Access to Drug and Food.**

| **Animal** | **ED50 Cocaine** | **ED50 Cocaine + Nicotine** |
| --- | --- | --- |
| F-7870 | 0.060 | 0.020 |
| F-7902 | 0.065 | 0.021 |
| F-8534 | 0.022 | 0.008 |
| F-7905 | 0.020 | 0.007 |
| F-7833 | 0.019 | 0.006 |
| F-8531 | 0.020 | 0.007 |
| **MEAN** | **0.034 ± 0.003** | **0.012 ± 0.003** |

Data are mean ± SEM.

**TABLE S3: ED50 Values for Individual Male Monkeys Responding Under Concurrent Access to Drug and Food**

| **Animal** | **ED50 Cocaine** | **ED50 Cocaine + Nicotine** |
| --- | --- | --- |
| M-7478 | 0.002 | 0.001 |
| M-8180 | 0.020 | 0.007 |
| M-8564 | 0.020 | 0.007 |
| M-8558 | 0.030 | 0.027 |
| M-8103 | 0.020 | 0.007 |
| M-8181 | 0.006 | 0.001 |
| M-8503 | 0.02 | 0.007 |
| M-7906 | 0.018 | 0.01 |
| **MEAN** | **0.017 ± 0.006** | **0.009 ± 0.003** |

Data are means ± SEM.

**TABLE S4: IP Values for Individual Monkeys Responding Under the Delay Discounting Procedure.**

| **Animal** | **IP Cocaine** | **IP Cocaine + Nicotine** |
| --- | --- | --- |
| F-8534 | 13.85 | 13.39 |
| M-7478 | 28.97 | 38.84 |
| M-8180 | 105 | 155.12 |
| M-8564 | 80.23 | 183.53 |
| M-8103 | 87.59 | 132.48 |
| M-8181 | 46.09 | 72.76 |
| M-8503 | 96.32 | 99.09 |
| F-7905 | 46.09 | 229.31 |
| F-7833 | 120 | 154.19 |
| M-7906 | 71.58 | 87.27 |
| **MEAN** | **69.57 ± 10.96** | **116.60 ± 21.11** |

Animal numbers starting with M are males and animal numbers starting with F are female animals. Data are means ± SEM.
